# Skin lesions in a 30-year-old male having smear-positive pulmonary tuberculosis

**DOI:** 10.4103/1817-1737.44784

**Published:** 2009

**Authors:** Prem P. Gupta, V. K. Jain, Dipti Agarwal, P. T. Yaseer

**Affiliations:** *Departments of Tuberculosis and Respiratory Medicine, Postgraduate Institute of Medical Science, Rohtak, India*; 1 *Departments of Skin and VD, Postgraduate Institute of Medical Science, Rohtak, India*; 2 *Department of Physiology, Postgraduate Institute of Medical Science, Rohtak, India*

A 30-year-old male, farm worker, chronic smoker presented at our institute with low-grade intermittent fever, cough with expectoration and asymptomatic, painless, dusky red skin lesions varying from the size of a peanut (3-–4 mm) to a coin (up to 3-–4 cm) over the upper trunk (back), neck and bilateral extensor surface of the upper limbs [Figures 1–5]. He had smear-positive pulmonary tuberculosis diagnosed in January 2006. He was given rifampicin, isoniazid, pyrazinamide and ethambutol for 2 months, by the end of which his sputum converted to negative, at which stage he was continued with rifampicin and isoniazid for a further 4 months. All drugs were supervised and administered thrice a week. After that, he remained symptom free for 2 years. At present, he is having an average build and good nutrition, with stable vital signs. There was no clubbing, cyanosis, ictenus or pallor. There was no enlargement of any group of lymph nodes. The liver was not palpable and the examination of other systems was not remarkable. The chest radiograph was suggestive of bilateral upper zone fibrocavitary disease, which was suggestive of tuberculosis. The sputum smear examination confirmed the presence of acid-fast bacilli. He was diagnosed as having smear-positive pulmonary tuberculosis and was started with WHO category II antitubercular treatment. A skin wedge biopsy was also taken.

**Figure 1:**
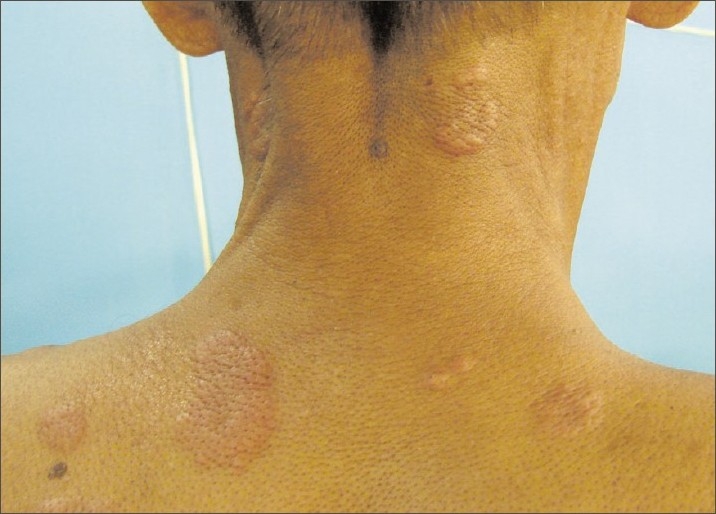
Multiple skin lesions along the back and neck

**Figure 2:**
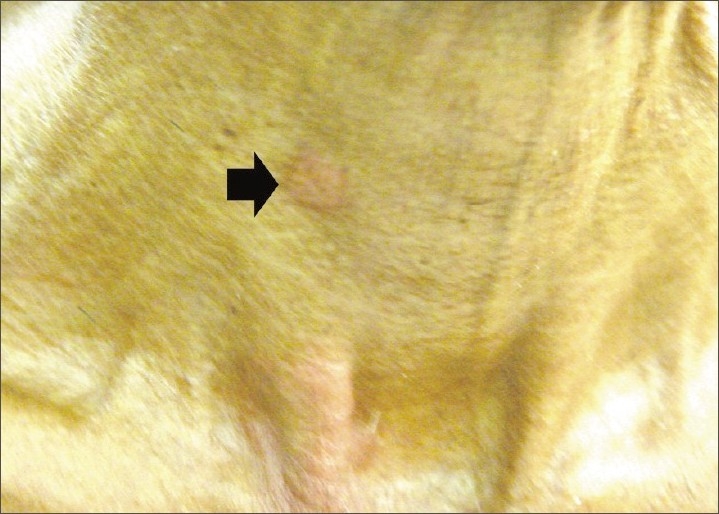
A characteristic lesion over the front aspect of the neck

**Figure 3:**
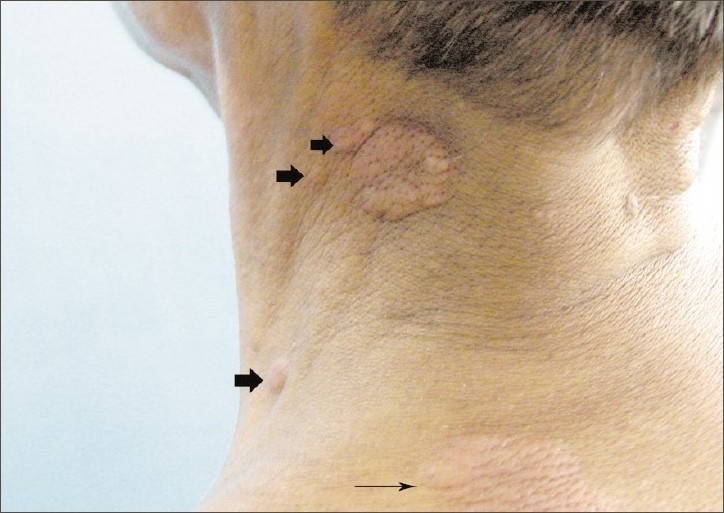
Oblique view showing skin lesions over the left side of the neck

**Figure 4:**
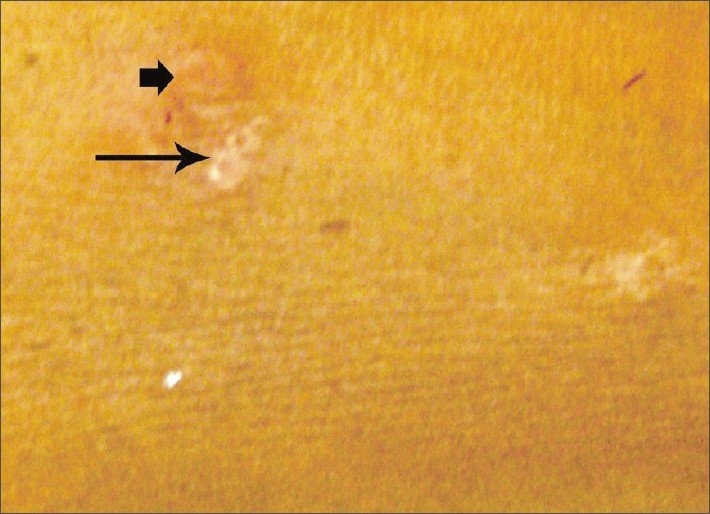
(Small thick arrow) papules with a central punctum; (big thin arrow) involuted lesion with pitted scars

**Figure 5:**
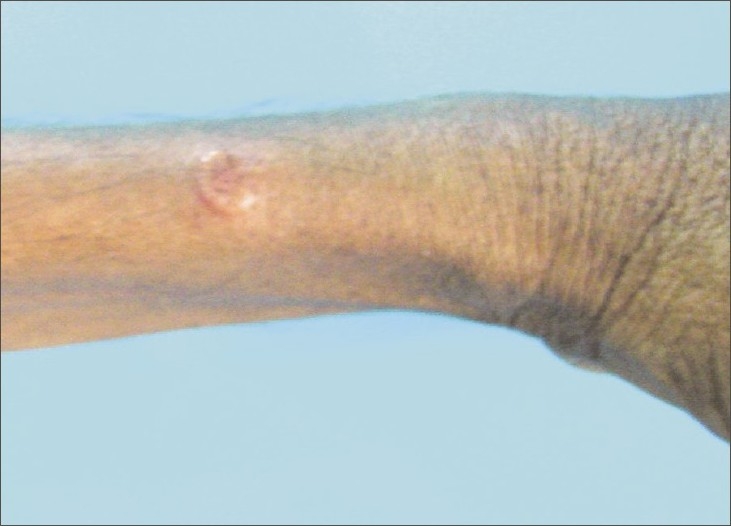
Left forearm: Papules with a central punctum

## Clinical Questions

1. What is the diagnosis of skin lesions?
2. Can you guess the immune status of this patient?
3. Is there any seasonal variation of this disease?
4. Can you demonstrate mycobacteria in skin lesion?

## Answers

1. Papulonecrotic tuberculid. Papulonecrotic tuberculid is a subgroup of tuberculid cutaneous disorders in which there is symmetric eruption of necrotizing papules appearing in crops and healing is with scar formation.[1] In present case, the papules showed a central depression and adherent crust and some had necrosis in the center. Skin biopsy showed a wedge-shaped necrosis of the upper dermis, which was extending to and involving the epidermis. A granulomatous lesion including the epitheloid cells and langerhans giant cells was seen. An obliterative endarteritis and endophlebitis was observed at places.
2. In this patient, the tuberculin test was strongly positive (an induration of 18 mm at 48 h and also at 72 h). Characteristically, patients with papulonecrotic tuberculid have good immune status and are in relatively good health. These patients show (1) positive tuberculin sensitivity, (2) tuberculous involvement (usually inactive) of viscera or lymph nodes but less commonly concomitant active disease, as is evident in the present case, (3) negative staining and culture for pathogenic mycobacteria in affected tissue and (4) skin lesions that heal with remission or treatment of TB.
3. Classically, papulonecrotic tuberculid is worse during the winter months.
4. As a rule, the isolation of the bacilli had been difficult in papulonecrotic tuberculid lesions. Some workers had been successful in recovering the organism.[2] The polymerase chain reaction (PCR) technique provides promising results by detecting mycobacterial DNA in the skin lesions. Few patients with histopathologically confirmed papulonecrotic tuberculid lesions had been studied[3] to detect *M. tuberculosis* DNA in the lesions using a gene amplification PCR. A 285-bp sequence specific for the *M. tuberculosis* complex was amplified and confirmed by Southern blot hybridization with a 32 p 5'-labeled internal probe.[3]

## Discussion

Although the global prevalence of tubercular infection remains very high and, in certain regions, the tubercular disease is a cause of great public health concern, cutaneous tuberculosis is seen in less than 0.1% of individuals attending dermatology clinics. In a survey[4] from dermatology clinics in Hong Kong, the incidence of cutaneous TB was 179 per 267,089 patients (0.07%); in northern India,[5] 0.1% of dermatology patients had cutaneous tuberculosis. Lupus vulgaris has been reported to be the most frequent form of skin manifestation (55%), followed by scrofuloderma (27%), TB verrucosa cutis (6%), tuberculous gumma (5%) and tuberculids (7%).[1] The term “tuberculids” is often applied to recurrent skin eruptions that are disseminated and symmetrical and have a predisposition of spontaneous involution. The earlier perception was that they were the outcome of reactions to toxins of tubercle bacilli and, hence, were not considered as“true” cutaneous tuberculosis. In recent times, they are believed to originate from a hematogenous dissemination of mycobacteria and a subsequent interplay of the number of bacilli and virulence as well as the host immune response determines the clinical course. The PCR technique makes the detection of mycobacterial DNA in tuberculids a reality and, therefore, provides a rational basis for antituberculous chemotherapy.

Papulonecrotic tuberculid is more frequent among children and the young adults. Two-thirds of the cases occur before the age of 30 years. Females are involved more frequently compared with males (3:1). The disease is known for its propensity to occur on the extensor surface of the extremities, buttocks and lower trunk in a symmetric distribution and often in clusters. They have been reported to involve the face and glans penis. Individual lesions are asymptomatic, small, dusky red papules, 3–5 mm, with a central punctum or crust. Involution is reported after 6–8 weeks, which leaves behind pitted scars. Histologically, these lesions show a wedge-shaped necrosis of the upper dermis involving the epidermis. Epithelioid cells as well as Langhans giant cells may be seen. An obliterative granulomatous vasculitis with fibrin in vessel walls and lumen is characteristic. Endovasculitis and occlusions of dermal blood vessels are protean histological features and, actually, represent the only fact known about the pathogenesis of this disorder.

The list of diseases that can be considered for differential diagnosis of papulonecrotic tuberculid is long and includes entities such as leukocytoclastic vasculitis, lymphomatoid papulosis, papular eczema, prurigo simplex with neurotic excoriation, pityriasis lichenoides et varioliformis acuta, lichen urticatus, Churg–Strauss granuloma and secondary syphilis. A thorough history, clinical appearance and histology are helpful to reach the diagnosis. The concomitant association of papulonecrotic tuberculid with pulmonary tuberculosis is rare but has been described.[6] The disease is characterized by a variable and unpredictable course because it may be chronic and recurrent and may present in successive crops at different sites even after antitubercular chemotherapy.
